# Evaluation of Suburethral Tissue Elasticity Using Strain Elastography in Women with Stress Urinary Incontinence

**DOI:** 10.3390/jcm14165617

**Published:** 2025-08-08

**Authors:** Lóránt Csákány, Zoltan Kozinszky, Flórián Kovács, Seron Kíra Krajczár, Szabolcs Várbíró, Attila Keresztúri, Gábor Németh, Andrea Surányi, Norbert Pásztor

**Affiliations:** 1 Department of Obstetrics and Gynecology, Albert Szent-Györgyi Medical School, University of Szeged, 6725 Szeged, Hungary; 2 Capio Specialized Center for Gynecology, Solna, 182 88 Stockholm, Sweden; 3 Institute of Plant Sciences and Environmental Protection, Faculty of Agriculture, University of Szeged, 6720 Szeged, Hungary; 4 Department of Agro-Environmental Studies, Hungarian University of Agriculture and Life Sciences, 1118 Budapest, Hungary; 5 Institute of Reproductive Medicine, Albert Szent-Györgyi Medical School, University of Szeged, 6723 Szeged, Hungary

**Keywords:** diagnostic imaging, female pelvic floor, introital ultrasound, pelvic floor dysfunction, strain elastography, stress urinary incontinence, suburethral stiffness, tissue biomechanics, ultrasound elastography, urethral support

## Abstract

**Objectives**: Strain elastography (SE) is a non-invasive ultrasound-based technique for evaluating tissue elasticity. This study investigated whether SE can reproducibly detect differences in suburethral tissue stiffness between women with stress urinary incontinence (SUI) and continent controls. **Methods**: In this prospective cohort study, 40 women (20 with SUI, 20 continent controls) underwent introital two-dimensional (2D) ultrasound in the midsagittal plane at rest. SE was performed at three predefined suburethral regions of interest (ROIs): the internal urethral orifice (IUO), midurethra (MU), and external urethral orifice (EUO), with the adipose layer (AL) serving as reference tissue. Group comparisons and reproducibility analyses were conducted. **Results**: SE enabled reliable in vivo assessment of suburethral elasticity. Women with SUI demonstrated significantly higher tissue elasticity at all three urethral levels compared to controls. The MU level showed the highest diagnostic accuracy (AUC = 0.813; sensitivity = 0.65; specificity = 0.85). Measurement reproducibility was excellent, with intraclass correlation coefficients exceeding 0.95 across all ROIs. **Conclusions**: SE is a feasible, reproducible imaging modality for assessing suburethral biomechanics in women with SUI. It effectively distinguishes affected individuals from continent controls, particularly at the midurethral level. Standardized protocols and diagnostic thresholds are needed to facilitate clinical integration of SE in the evaluation and management of SUI.

## 1. Introduction

Urinary incontinence is a common condition that negatively impacts women’s quality of life, daily activities, and psychosocial well-being. Among its subtypes, stress urinary incontinence (SUI) is the most prevalent, affecting up to 25% of women. According to the International Continence Society (ICS), SUI is defined as the involuntary loss of urine during physical exertion, sneezing, or coughing, typically resulting from incomplete urethral closure during episodes of elevated intra-abdominal pressure [1,2,3,4].

SUI has a multifactorial etiology, including insufficient bladder neck support and reduced stabilization by paraurethral connective tissues, often associated with connective tissue laxity [2,4,5,6]. Urethral hypermobility is a key pathophysiological feature of SUI and may result from compromised suburethral support. According to the “hammock hypothesis”, continence is maintained by a supportive layer beneath the urethra—comprising the anterior vaginal wall, endopelvic fascia, arcus tendineus fascia pelvis, and levator ani muscle—that compresses the urethra during increases in intra-abdominal pressure [1,6,7]. If the structural or biomechanical integrity of these tissues is compromised—for example, due to extracellular matrix degradation—urethral support may be weakened, resulting in hypermobility and reduced closure pressure [8,9,10,11].

While introital two-dimensional (2D) ultrasound is widely used to evaluate urethral hypermobility, it provides no information on the biomechanical properties of periurethral tissues [12,13]. Similarly, three-dimensional (3D)/four-dimensional (4D) transperineal ultrasound and magnetic resonance imaging (MRI) enable detailed visualization of structural abnormalities—such as levator ani muscle avulsion or intrinsic sphincter deficiency—but do not allow for the direct assessment of tissue stiffness [4,7,14,15].

This lack of biomechanical insight has prompted growing interest in ultrasound elastography, particularly strain elastography (SE), as a non-invasive method for characterizing tissue elasticity in vivo. SE is a qualitative and semi-quantitative imaging modality that assesses tissue deformation in response to externally applied compression [16,17].

SE has been successfully applied in various gynecological conditions—including endometriosis, polycystic ovary syndrome, uterine fibroids, and endometrial polyps—demonstrating added diagnostic value when used alongside conventional ultrasound [12,16,17,18,19,20].

Despite these promising applications, the role of SE in evaluating the biomechanical properties of pelvic floor support structures remains largely unexplored. To date, only one study has attempted to investigate the association between paraurethral tissue elasticity and urethral mobility using SE, but it yielded inconclusive results [17].

Given the limited evidence regarding the use of SE for assessing suburethral support in SUI, we hypothesized that SE could distinguish women with SUI from continent controls by characterizing differences in tissue elasticity. This study aimed to evaluate whether SE can reliably detect variations in suburethral tissue stiffness between these two groups.

## 2. Materials and Methods

### 2.1. Study Participants

This prospective observational cohort study was conducted between 16 August 2024, and 1 January 2025, at the Department of Obstetrics and Gynecology, University of Szeged, Hungary. Consecutive women referred for urogynecological evaluation were assessed by a subspecialist in urogynecology (L.Cs.) with over five years of clinical experience. Twenty women with clinically confirmed pure SUI were enrolled and compared with twenty healthy continent women who served as controls. SUI was diagnosed based on a history of involuntary urine leakage during episodes of increased intra-abdominal pressure, confirmed by a positive cough stress test with a comfortably full bladder (≥200 mL), in accordance with ICS criteria [21].

Exclusion criteria were as follows: age under 35 years, presence of pelvic organ prolapse, mixed urinary incontinence, prior vaginal reconstructive or urethral surgery, history of bladder augmentation, post-void residual volume >150 mL, active smoking, and known connective tissue disorders. Participants with technically limited ultrasound imaging due to body habitus were also excluded. Additional exclusion criteria included hormonal therapy within the past 12 months, untreated urinary tract infection, current malignancy, psychiatric illness, or pregnancy.

An a priori power analysis was conducted using G*Power (version 3.1.9.7; Heinrich Heine University, Düsseldorf, Germany) to determine the required sample size for this pilot study evaluating the diagnostic performance of SE in women with SUI. In the absence of previously reported effect sizes, a moderate effect (Cohen’s d = 0.7) was assumed. Using a two-tailed α = 0.05 and β = 0.2 (corresponding to 80% statistical power), the analysis indicated that 20 participants per group were sufficient to detect statistically significant differences [22].

### 2.2. Ultrasound Examination

All introital ultrasound examinations were performed using a GE Voluson S10 system (BT18) equipped with a GE RIC5-9A-RS (GE HealthCare Austria GmbH & Co OG, Tiefenbach, Austria) transvaginal probe. First, the lower urinary tract was assessed for anatomical integrity, including evaluation of urethral length, funneling, and mobility. Subsequently, SE was performed to assess suburethral tissue elasticity and structural support. All examinations were conducted by a single experienced urogynecologic sonographer (A.S.), who was blinded to participants’ clinical and symptom data.

### 2.3. Introital Sonography

The transducer was positioned at the level of the external urethral orifice and aligned parallel to the body axis. Examinations were performed with participants in the supine position, knees slightly flexed, and bladder emptied.

Minimal probe pressure was applied to avoid compressing suburethral structures, displacing the bladder neck, or distorting urethral dynamics [13].

On midsagittal B-mode imaging, the urethra appeared in a retropubic position and perpendicular to the pelvic floor at rest and during strain. The entire urethra, from the bladder neck to the external meatus, along with the pubic symphysis and anterior vaginal wall, was visualized to assess urethral funneling and hypermobility [13,23,24].

Bladder neck mobility was quantified relative to the inferoposterior margin of the pubic symphysis; a Valsalva-induced descent >10 mm was considered indicative of hypermobility [25].

### 2.4. Strain Elastography Protocol

SE was employed to assess relative tissue stiffness as an indirect indicator of elasticity. SE images were obtained by applying controlled manual compression using a transvaginal probe to induce tissue deformation [26]. In general, softer tissues deform more than stiffer tissues under external pressure; the degree of deformation reflects the underlying mechanical properties of the tissue, with greater displacement indicating lower stiffness and, conversely, greater elasticity [16].

To ensure optimal image quality and reduce signal artifacts, precompression was minimized by maintaining only gentle contact between the probe and the vaginal wall [27].

**Figure 1:**
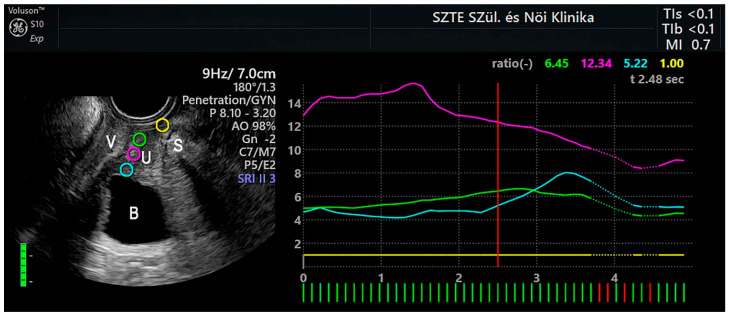
Anatomical locations of the regions of interest (ROIs) with corresponding strain elastography (SE) measurements. Key anatomical landmarks include the vagina (V), urethra (U), bladder (B), and symphysis pubis (S). The blue ROI circle marks the tissue surrounding the internal urethral orifice, the purple ROI circle indicates the endopelvic fascia at the midurethral level, the green ROI circle shows the tissue adjacent to the external urethral orifice (EUO), and the yellow ROI circle represents the adipose layer between the EUO and the symphysis pubis. The vertical red line on the strain graph indicates the selected timepoint at which the displayed strain ratios were measured. In the lower left corner, a standardized six-point image quality score is displayed, providing an objective assessment of the SE signal quality.

During image acquisition, the probe was held steadily for at least 5 s with minimal movement, following the method described by Saliha et al. [18]. Tissue displacement was monitored by tracking speckle pattern changes before and after compression. Simultaneously, a 2D B-mode image of the suburethral region and the corresponding strain elastogram were acquired (Figure 1). Tissue stiffness variations were visualized in real time as color-coded elastograms, enabling dynamic adjustment of compression based on a built-in strain indicator bar.

All images were acquired at rest to prevent levator ani muscle contraction and involuntary voiding. To evaluate suburethral elasticity at anatomically defined locations, three manually placed, 5 mm circular regions of interest (ROIs) were positioned between the urethra and anterior vaginal wall at predefined proximal, mid-, and distal urethral landmarks.

Real-time SE combined with B-mode introital 2D ultrasound for suburethral tissue elasticity assessment. Dual-panel image showing midsagittal B-mode ultrasound (left) and corresponding SE output (right). Four color-coded, anatomically defined ROIs were assessed: blue—tissue surrounding the internal urethral orifice; purple—endopelvic fascia at the midurethral level; green—tissue adjacent to the external urethral orifice (EUO); and yellow—the adipose layer between the EUO and the symphysis pubis, used as reference tissue. Strain values were continuously tracked and displayed as real-time curves on the right panel, with curve colors corresponding to their respective ROI color codes.

Image quality was rated using a standardized six-point scale, and only images scoring 5 or 6—representing optimal compression—were included in the analysis. This approach ensured reproducible image quality, as both excessive and insufficient probe pressure can compromise elastogram interpretability.

Although the magnitude of applied pressure was not quantitatively measured, this limitation does not compromise clinical utility, as SE intrinsically provides relative, rather than absolute, measurements of tissue stiffness [16,17].

### 2.5. Regions of Interest (ROIs) Placement

Color-coded ROIs were defined according to distinct anatomical landmarks as follows (Figure 1).

#### 2.5.1. Yellow ROI: Adipose Layer (AL)

The yellow ROI was placed within the AL layer located between the external urethral meatus and the pubic symphysis. This site served as a reference region, representing the softest tissue in the field of view due to its high adipocyte content and lack of involvement in urethral support [28,29].

#### 2.5.2. Blue ROI: Internal Urethral Orifice (IUO) Level

The blue ROI was positioned at the level of the IUO, anatomically corresponding to the bladder neck. This area comprises transitional urothelium, lamina propria, and smooth muscle fibers continuous with the detrusor muscle, collectively forming the internal urethral sphincter (IUS). Located at the bladder base and proximal urethra, the IUS contributes to continence by maintaining closure during elevations in intra-abdominal pressure [5,29,30].

#### 2.5.3. Purple ROI: Midurethral (MU) Level

The purple ROI was placed at the MU level, at the interface between the urethral wall and the adjacent endopelvic connective tissue. This region is critical for urethral support, where the levator ani muscle and endopelvic fascia act together to counteract increases in intra-abdominal pressure. Histological studies describe this area as a well-organized fibromuscular complex that transmits force to compress the urethra against its supportive layer, thereby contributing to urethral closure [5,24,29,30,31].

#### 2.5.4. Green ROI: External Urethral Orifice (EUO) Level

The green ROI was placed at the distal urethra, between the EUO and the anterior vaginal wall. This region consists primarily of loose fibrous connective tissue [5,29,30].

ROI placement followed a standardized anatomical protocol to ensure systematic and reproducible assessment of tissue elasticity at three distinct suburethral levels. In our cohort, the mean urethral length was 30.8 mm, which falls within the reported physiological range of 19 to 45 mm for the female urethra [32].

To assess measurement repeatability, each ROI was evaluated five times, and the peak strain values were recorded to ensure reproducibility.

### 2.6. Statistical Evaluation

All statistical analyses were performed using R software (version 4.2.1) [33]. Categorical variables are presented as frequencies and percentages (n, %), while continuous data are expressed as mean ± standard deviation (SD). To describe the distribution of ultrasound-derived measurements within the SUI and control groups, additional descriptive statistics—including skewness and kurtosis—were calculated. The normality of residuals derived from SE values was assessed using the Kolmogorov–Smirnov test.

Between-group comparisons were conducted using the chi-square test for categorical variables, the independent samples *t*-test for normally distributed continuous variables, and the Wilcoxon rank-sum test for non-normally distributed data.

Measurement reliability was assessed using intraclass correlation coefficients (ICCs) and Cronbach’s alpha [34]. Values exceeding 0.90 were interpreted as indicative of excellent reproducibility and internal consistency [35]. To evaluate differences in mean SE values across repeated measurements at each ROI, a multivariate analysis of variance (MANOVA) was performed, adjusting for age and body mass index (BMI) at the IUO, MU, and EUO levels. Wilks’ lambda was used to determine overall model significance, and Levene’s test was applied to assess homogeneity of variances. Where significant differences were identified, post hoc comparisons were conducted using the Games–Howell test.

All statistical tests were two-sided, and a *p*-value of <0.05 was considered statistically significant.

To evaluate diagnostic accuracy, receiver operating characteristic (ROC) curve analysis was performed for each ROI, and the corresponding area under the curve (AUC) was calculated [34]. AUC values above 0.90 were interpreted as demonstrating strong discriminatory ability [36]. Additional performance metrics—including sensitivity, specificity, positive predictive value (PPV), negative predictive value (NPV), and F1 score [37]—were also calculated to comprehensively evaluate the classification performance of SE.

Trial Registration: Elastographic Assessment of Suburethral Tissue in Continent and Incontinent Women (SE-inc1), NCT06933407, 16.08.2024, https://clinicaltrials.gov/study/NCT06933407 (accessed on 5 August 2025).

## 3. Results

### 3.1. Demographic and Clinical Characteristics

Participant characteristics are summarized in Table 1. Women with SUI exhibited significantly higher body weight and BMI compared to continent controls. No statistically significant differences were found between groups in terms of age, parity, or postmenopausal status. Urethral length was comparable across groups.

Signs of urethral dysfunction were more frequent among participants with SUI: urethral hypermobility was observed in 12 individuals (60.0%), and urethral funneling was detected in 8 individuals (40.0%) during the Valsalva maneuver.

### 3.2. Strain Elastography Measurements

At the level of the EUO in the control group, SE measurements did not meet the assumption of normality. In contrast, SE data from all other ROIs demonstrated a Gaussian distribution. A detailed summary of distribution characteristics is provided in the Supplementary Materials (Table S1).

Multivariate analysis of variance (MANOVA) across the three anatomical levels revealed a significant group effect (Wilks’ lambda = 0.588, F(3, 35) = 8.189, *p* < 0.001), indicating statistically significant differences in suburethral tissue elasticity between women with SUI and continent controls.

**Figure 2:**
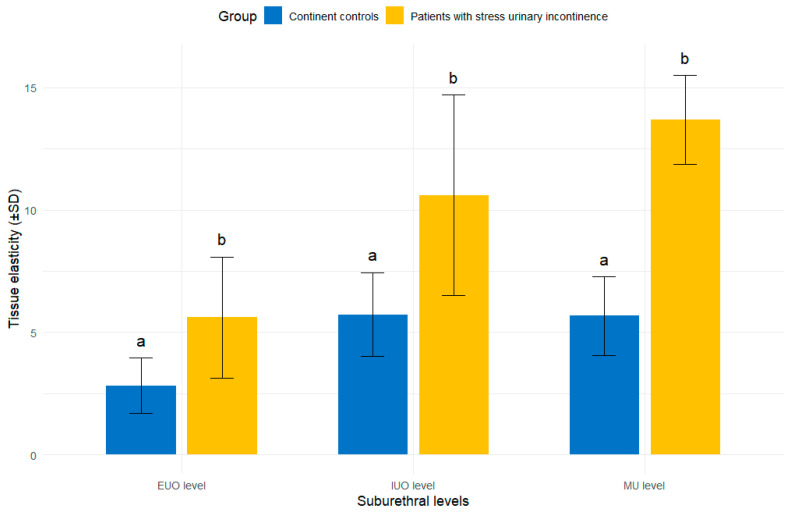
Comparison of suburethral tissue elasticity between continent controls and women with stress urinary incontinence.

At all urethral levels, participants with SUI exhibited significantly higher strain values compared to continent controls (Games–Howell post hoc test, *p* < 0.05), indicating lower suburethral tissue stiffness. Mean SE values (mean ± SD) in the SUI vs. control groups were 10.60 ± 8.19 vs. 5.73 ± 3.45 at the IUO, 13.68 ± 7.24 vs. 5.67 ± 3.21 at the MU, and 5.61 ± 4.95 vs. 2.82 ± 2.26 at the EUO. These findings confirm a consistent increase in tissue deformability across all suburethral regions in women with SUI. Furthermore, greater inter-individual variability was observed in the SUI group (Figure 2).

This was further supported by a post hoc analysis using the actual observed values at the midurethral level (mean = 13.68, SD = 5.6 vs. null hypothesis value = 5.67), which yielded a minimum required sample size of only 7 participants and an observed Cohen’s d = 1.75, indicating a very large effect size and strong statistical power.

Mean SE values (±SD) are presented for three anatomically defined ROIs: the internal urethral orifice (IUO), midurethra (MU), and external urethral orifice (EUO), based on five repeated measurements per participant. Bar heights represent mean elasticity values; error bars indicate standard deviations. Different lowercase letters above the bars denote statistically significant between-group differences, as determined by the Games–Howell post hoc test (*p* < 0.05).

All five repeated SE measurements per ROI demonstrated statistically significant differences between groups, consistently indicating increased elasticity in the SUI cohort. Minor exceptions were observed at the IUO level and in one of the five repetitions at the EUO level, where group differences did not reach statistical significance (Table S2).

### 3.3. Reproducibility of Strain Elastography Measurements

The reliability of repeated SE measurements was assessed at three predefined anatomical levels: IUO, MU, and EUO, corresponding to the blue, purple, and green ROIs, respectively (Figure 1).

Internal consistency, evaluated using Cronbach’s alpha, was excellent across all measurement sites in both the SUI and control groups. At the IUO level, alpha coefficients were 0.96 in both groups, with minimal variation after data standardization (0.97 in SUI; 0.96 in controls). Similarly, high internal consistency was observed at the MU level (controls: 0.98; SUI: 0.95), which remained stable after standardization. At the EUO level, Cronbach’s alpha values reached 0.98 in controls and 0.99 in the SUI group—the highest observed across all anatomical levels. All alpha values exceeded 0.95, indicating excellent internal reliability.

These findings were corroborated by intraclass correlation coefficient (ICC) analysis, which further demonstrated strong reproducibility. At the IUO level, ICCs were 0.969 (95% CI: 0.941–0.986) in controls and 0.962 (95% CI: 0.927–0.983) in the SUI group. At the MU level, values were 0.983 (95% CI: 0.968–0.993) in controls and 0.954 (95% CI: 0.913–0.980) in the SUI group. At the EUO level, ICCs reached 0.978 (95% CI: 0.957–0.990) in controls and 0.991 (95% CI: 0.982–0.996) in the SUI group.

Together, these metrics confirm a high degree of measurement reliability and internal consistency across all suburethral ROIs in both cohorts, supporting the reproducibility of SE for evaluating suburethral tissue stiffness (Table 2).

### 3.4. Diagnostic Performance of Strain Elastography in Stress Urinary Incontinence

**Figure 3:**
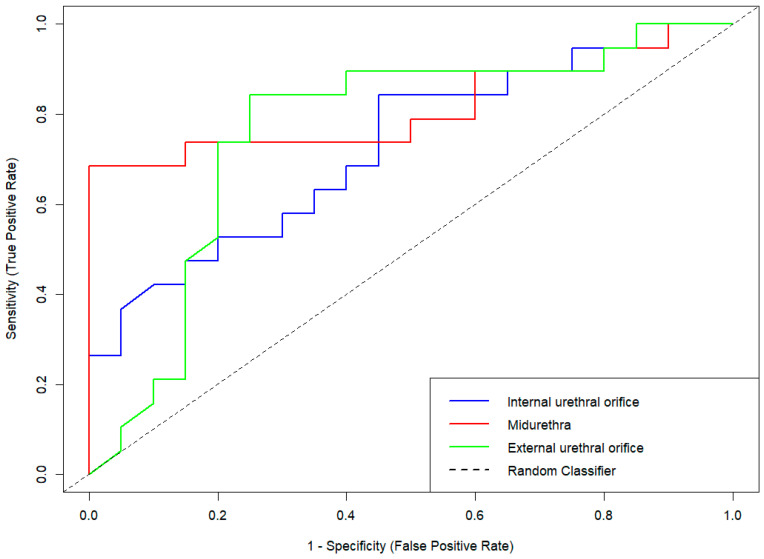
Receiver operating characteristic (ROC) curves for strain elastography (SE) across suburethral regions of interest (ROIs) in women with stress urinary incontinence.

ROC curve analysis was performed to evaluate the diagnostic performance of SE across the three predefined anatomical ROIs. Among these, the MU level demonstrated the highest discriminatory capacity, with an area under the curve (AUC) of 0.813 (95% CI: 0.666–0.960, *p* < 0.001). The EUO showed an AUC of 0.763 (95% CI: 0.603–0.924, *p* < 0.01), while the IUO yielded the lowest AUC at 0.728 (95% CI: 0.569–0.886, *p* < 0.05) (Figure 3).

The diagnostic performance of SE measurements was evaluated across three anatomical ROIs: internal urethral orifice (IUO), midurethra (MU), and external urethral orifice (EUO). The dashed diagonal line indicates the line of no discrimination (random classifier). Among the three ROIs, the MU region demonstrated the highest diagnostic accuracy based on the area under the curve (AUC), followed by the EUO and IUO.

Diagnostic performance metrics are summarized in Table 3. The MU region exhibited the most favorable diagnostic profile, with a sensitivity of 0.65 and a specificity of 0.85. In comparison, the IUO region demonstrated the lowest sensitivity (0.58) and moderately reduced specificity (0.70), whereas the EUO showed intermediate values (sensitivity: 0.60; specificity: 0.80).

Positive predictive value (PPV) and negative predictive value (NPV) were also highest at the MU level (PPV = 0.81; NPV = 0.71), indicating improved classification performance for both SUI and continent individuals. The EUO region showed moderately high predictive values (PPV = 0.75; NPV = 0.67), while the IUO level yielded the lowest (PPV = 0.65; NPV = 0.64).

Balanced accuracy—defined as the mean of sensitivity and specificity—was highest at the MU level (0.75), followed by EUO (0.70) and IUO (0.64). These findings are consistent with the corresponding AUC values, further reinforcing the superior diagnostic performance of the MU region.

Finally, evaluation of the F1 score—a harmonic mean of precision and recall—revealed the highest value at the MU level (0.72), outperforming the EUO (0.67) and IUO (0.61). Collectively, these results confirm that the midurethral segment is the most diagnostically informative ROI for distinguishing women with SUI from continent controls.

## 4. Discussion

SUI resulting from impaired urethral support is a highly prevalent condition that significantly compromises women’s quality of life by limiting physical activity, emotional well-being, and social participation [2,23,38,39]. Accordingly, functional imaging of the pelvic floor plays a central role in urogynecologic diagnostics. While perineal ultrasound remains the most widely used modality, its anatomically oriented approach limits the assessment of soft tissue mechanical properties that are critical for maintaining continence [15,23,24,31,39].

SE is a non-invasive, real-time ultrasound-based technique that enables in vivo assessment of tissue elasticity. In recent years, SE has emerged as a promising modality for functional pelvic floor imaging, offering a biomechanical perspective that complements traditional anatomical approaches [16,17,31].

To date, only one prior study—by Kreutzkamp et al.—has assessed suburethral elasticity using SE. Unlike our results, that study found no association between elasticity and incontinence. However, several methodological limitations may explain this discrepancy. Their cohort included women with mixed types of incontinence, potentially obscuring group-level differences. Only two closely spaced ROIs were examined, without anatomical standardization or reference tissue normalization. Additionally, reproducibility was not evaluated, limiting interpretability and comparability.

Building on these limitations, our study is the first to assess suburethral tissue stiffness using SE at three anatomically defined urethral levels, with normalization to adjacent periurethral adipose tissue. A well-characterized cohort was examined using a structured protocol with ROI placement at the IUO, MU, and EUO. The primary aim was to evaluate the reproducibility and diagnostic utility of introital SE in distinguishing isolated SUI from continence.

Consistent with our hypothesis, the results revealed significantly increased suburethral elasticity in women with SUI, indicating reduced tissue stiffness within the urethral supportive structures. Among the three measured levels, the midurethral ROI showed the highest diagnostic accuracy, underscoring its central role in urethral closure. This finding aligns with the pathophysiological concept—reflected in DeLancey’s “hammock hypothesis”—that a firm suburethral backboard formed by the endopelvic fascia and anterior vaginal wall is essential for effective pressure transmission [1,29,31]. When these connective tissues are lax or poorly anchored, urethral closure fails under stress, resulting in urinary leakage [1,7,23,31].

Suburethral tissue weakening in SUI may partly originate from childbirth-related trauma. Vaginal delivery is associated with a threefold higher risk of SUI compared to cesarean section, primarily due to injuries affecting urethral support and sphincteric integrity. In nulliparous women with de novo SUI, reduced maximal urethral closure pressure and increased bladder neck mobility reflect combined impairment of anatomical and functional support. Trauma to the pelvic floor muscles—particularly the levator ani—may also compromise adjacent structures such as the paraurethral tissues, further exacerbating dysfunction [15,39].

Importantly, these diagnostic differences were accompanied by robust measurement reproducibility. In our study, SE demonstrated excellent reliability across all suburethral levels, with the highest ICC observed at the EUO in the SUI group. Internal consistency, assessed by Cronbach’s alpha, also exceeded 0.95 at all regions of interest in both cohorts, underscoring the methodological reliability of the technique.

Despite the encouraging findings, certain limitations must be acknowledged. First, this was a single-center pilot study with a relatively small sample size, which may restrict generalizability. Second, although intra-observer reproducibility was excellent, inter-observer agreement and cross-platform validation were not assessed.

Complementary findings have emerged from studies using shear wave elastography (SWE) to assess pelvic floor biomechanics. Li et al. investigated the perineal body (PB) and reported no significant stiffness differences at rest; however, Valsalva-induced stiffening was markedly attenuated in women with SUI, suggesting that PB dysfunction may only manifest under mechanical load [39]. In contrast, our study demonstrated increased suburethral elasticity at rest in women with SUI, indicating baseline tissue weakness independent of provocation. Our results are further supported by the recent SWE study by De Vicari et al., which detected significant differences in urethral elasticity and bladder neck mobility between SUI patients and continent controls [38]. Taken together, these complementary findings underscore the critical role of paraurethral tissues in maintaining continence and suggest that biomechanical impairment in SUI is not limited to the suburethral region but may also involve other key components of the continence mechanism, including the PB.

In summary, our findings demonstrate that SE is a feasible, reproducible, and anatomically standardized imaging modality for assessing suburethral support in women. By visualizing in vivo biomechanical deficits, SE provides clinically relevant functional insights that may inform individualized diagnosis, treatment planning, and longitudinal monitoring in SUI.

## 5. Conclusions

Based on our findings, SE can reliably differentiate women with SUI from continent controls—particularly at the midurethral level, where the most pronounced stiffness differences were observed. The use of anatomically predefined ROIs with normalization to adjacent reference tissue enabled excellent reproducibility of suburethral measurements. To the best of our knowledge, this is the first study to demonstrate significant differences in suburethral tissue elasticity between women with SUI and continent controls using SE. Given its diagnostic accuracy, methodological robustness, and clinical feasibility, SE shows strong potential as a non-invasive imaging tool for the assessment and individualized management of SUI.

## Figures and Tables

**Table 1:** Sociodemographic and clinical characteristics of study participants. Continuous variables are presented as mean ± SD; categorical variables are reported as frequency and percentage, n (%).

Variable	SUI ** (*n* = 20)		Continent Controls (*n* = 20)		*p*-Value
Age (years)	57.50 ± 12.60		54.15 ± 12.88		NS
Weight (kg)	82.95 ± 17.46		71.40 ± 16.72		0.03
BMI (kg/m^2^)	30.30 ± 5.02		26.56 ± 6.16		0.02
Number of pregnancies	2.30 ± 1.08		2.05 ± 1.36		NS
Number of deliveries	2.05 ± 0.94		1.60 ± 0.88		NS
Urethral length (mm)	3.17 ± 0.60		3.00 ± 0.46		NS
Postmenopausal status, n (%)	15 (no)	75.0 (yes)	11 (no)	55.0 (yes)	NS

** Patients with stress urinary incontinence. *p*-Values were calculated using the χ^2^ test for categorical variables and the unpaired *t*-test or Wilcoxon rank-sum test for continuous variables, as appropriate.

**Table 2:** Reproducibility of strain elastography measurements at each region of interest, based on intraclass correlation coefficients (ICC) (ICC values are presented with 95% confidence intervals and *p*-values for repeated measures).

Group	Internal Urethral Orifice (IUO)			Midurethra (MU)			External Urethral Orifice (EUO)		
	ICC	95% CI	*p*-Value	ICC	95% CI	*p*-Value	ICC	95% CI	*p*-Value
Continent controls	0.96	0.94–0.98	<0.001	0.98	0.96–0.99	<0.001	0.97	0.95–0.99	<0.001
SUI **	0.96	0.92–0.98	<0.002	0.95	0.913–0.98	<0.002	0.99	0.98–0.99	<0.002

** Patients with stress urinary incontinence.

**Table 3:** Diagnostic performance metrics of strain elastography across anatomically defined regions of interest.

Categories	IUO	MU	EUO
Sensitivity or Recall $\frac{TP_{i}}{TP_{i}+FN_{i}}$	0.58	0.65	0.60
Specificity $\frac{TN_{i}}{TN_{i}+FP_{i}}$	0.70	0.85	0.80
Positive Predictive Value $PPV_{i}=\frac{TP_{i}}{TP_{i}+FP_{i}}$	0.65	0.81	0.75
Negative Predictive Value $NPV_{i}=\frac{TN_{i}}{TN_{i}+FN_{i}}$	0.64	0.71	0.67
Prevalence $\frac{TP_{i}+FN_{i}}{N}$	0.49	0.50	0.50
Detection Rate $\frac{TP_{i}}{N}$	0.28	0.33	0.30
Detection Prevalence $\frac{TP_{i}+FP_{i}}{N}$	0.44	0.40	0.40
Balanced Accuracy $\frac{Sensitivity_{i}+Specificity_{i}}{2}$	0.64	0.75	0.70
Area Under the ROC Curve	0.72	0.81	0.76
F1 score $\frac{2\cdot PPV\cdot Sensitivity}{PPV+Sensitivity}$	0.61	0.72	0.67

Abbreviations: FN = false negative; FP = false positive; N = number of participants; NPV = negative predictive value; PPV = positive predictive value; ROC = receiver operating characteristic; TN = true negative; TP = true positive.

## Data Availability

The data that support the findings of this study are available from the corresponding author upon reasonable request.

## Supplementary Materials

The following supporting information can be downloaded at: https://www.mdpi.com/article/10.3390/jcm14165617/s1, Figure S1. Intraclass correlation coefficients (ICCs) for repeated strain elastography measurements at the internal urethral orifice level, shown separately for continent controls and women with stress urinary incontinence. Abbreviation: meas = measurement. Figure S2. Intraclass correlation coefficients (ICCs) for repeated strain elastography measurements at the midurethral level, shown separately for continent controls and women with stress urinary incontinence. Abbreviation: meas = measurement. Figure S3. Intraclass correlation coefficients (ICCs) for repeated strain elastography measurements at the external urethral orifice level, shown separately for continent controls and women with stress urinary incontinence. Abbreviation: meas = measurement. Table S1. Distribution and normality of strain elastography values across urethral regions of interest (ROIs) in women with stress urinary incontinence versus continent controls. Table S2. Strain elastography (SE) measurements across anatomically defined regions of interest (ROIs) in study participants.

## Abbreviations

The following abbreviations are used in this manuscript:

2D	two-dimensional
AL	adipose layer
AUC	area under the curve
BMI	body mass index
EUO	external urethral orifice
FN	false negative
FP	false positive
GE	General Electric
ICC	intraclass correlation coefficient
IUS	internal urethral sphincter
IUO	internal urethral orifice
LA	levator ani
MANOVA	multivariate analysis of variance
Meas	measurement
MRI	magnetic resonance imaging
MU	midurethra
N	number of participants
NPV	negative predictive values
NS	not significant
PB	perineal body
PPV	positive predictive values
ROI	region of interest
ROC	receiver operating characteristic
SD	standard deviation
SE	strain elastography
SUI	stress urinary incontinence
TN	true negative
TP	true positive
UI	urinary incontinence
US	ultrasound
