# Supplementary material for: Evaluation of Suburethral Tissue Elasticity Using Strain Elastography in Women with Stress Urinary Incontinence
Source: J Clin Med. 2025 Aug 8;14(16):5617. doi: 10.3390/jcm14165617 (PMC12386704; doi:10.3390/jcm14165617)
Supplement: Supplementary file 1 [file jcm-14-05617-s001.zip › jcm-3767994-supplementary.pdf]

Supplementary Materials

Kolmogorov–Smirnov tests confirmed that the assumption of normality was satisfied for SE data at the IUO level in both the control ($p > 0.05$) and SUI groups ($p > 0.05$). Similarly, SE values at the MU level followed a normal distribution in both cohorts ($p > 0.05$). In contrast, at the EUO level, the control group exhibited a statistically significant deviation from normality ($p < 0.05$), while the SUI group conformed to normal distribution ($p > 0.05$).

Descriptive statistics further supported these findings. At the IUO level, the SUI group demonstrated higher skewness (2.24) and kurtosis (6.80) compared to the control group (skewness: 0.88; kurtosis: 0.52). At the EUO level, the control group showed elevated skewness (1.65) and kurtosis (1.95), while the SUI group values were closer to normality (skewness: 0.41; kurtosis: –0.11). These results indicate that normality assumptions were met across all datasets, except for the EUO measurements in the control group (Table S1).

Regarding measurement reproducibility, ICCs across the five repeated SE acquisitions at each ROI exceeded 0.80 in most cases (Figure S1–S3). While repeated-measures ANOVA revealed significant intra-group variability at the IUO level in the control group ($p = 0.031$) and at the MU level in the SUI group ($p = 0.003$), ICC values remained consistently high (IUO: 0.78–0.91; MU: 0.71–0.92). These observations suggest that the detected variability reflects inherent physiological heterogeneity rather than compromised repeatability (Table S1).

Table S1. Distribution and normality of strain elastography values across urethral regions of interest (ROIs) in women with stress urinary incontinence versus continent controls.

Anatomical ROIs		Kolmogorov–Smirnov test			Descriptives		
		Statistic	p-value	result	Skewness	Kurtosis	median
IUO	Controls	0.182	0.098	TRUE	0.882	0.528	4.5780
	SUI**	0.171	0.175	TRUE	2.240	6.808	9.1020
MU	Controls	0.092	0.2	TRUE	0.437	-0.442	5.2800
	SUI**	0.182	0.118	TRUE	-0.177	-0.659	15.1000
EUO	Controls	0.245	0.004	FALSE	1.647	1.948	1.7040
	SUI**	0.105	0.2	TRUE	0.408	-0.112	4.2000

** Patients with stress urinary incontinence.

Abbreviations: EUO = external urethral orifice; IUO = internal urethral orifice; MU = midurethra.

Table S2. Strain elastography (SE) measurements across anatomically defined regions of interest (ROIs) in study participants.*

	SUI** (Mean ± SD)	Continent controls (Mean ± SD)	p-value	95% CI
SE measurements at the level of IUO††				
Measurement 1	6.52 ± 4.21	10.98 ± 9.14	0.06	−9.08–0.16
Measurement 2	5.64 ± 3.99	10.19 ± 8.12	0.03	−8.65–−0.45
Measurement 3	5.3 ± 3.27	10.93 ± 8.88	0.01	−9.99–−1.26
Measurement 4	5.31 ± 3.17	9.01 ± 7.88	0.06	−7.55–0.15
Measurement 5	6.14 ± 3.46	9.98 ± 9.08	0.09	−8.33–0.64
SE measurements at the level of MU††				
Measurement 1	5.92 ± 3.50	14.29 ± 8.68	<0.001	−12.68–−4.06
Measurement 2	5.60 ± 3.23	12.67 ± 7.21	<0.001	−10.70–−3.44
Measurement 3	5.45 ± 3.37	13.38 ± 7.59	<0.001	−11.75–−4.12
Measurement 4	5.35 ± 3.06	12.59 ± 7.74	0.001	−11.08–−3.41
Measurement 5	6.01 ± 3.38	13.53 ± 8.18	0.001	−11.59–−3.45
SE measurements at the level of EUO††				
Measurement 1	3.28 ± 2.74	5.39 ± 4.12	0.06	−4.34–0.13
Measurement 2	2.95 ± 2.20	5.58 ±5.42	0.05	−5.28–0.02
Measurement 3	2.76 ± 2.34	5.47 ± 4.82	0.03	−5.13–−0.28
Measurement 4	2.39 ± 2.11	5.29 ± 5.61	0.04	−5.61–−0.19
Measurement 5	2.73 ± 2.27	5.51 ± 4.74	0.02	−5.16–−0.40

* All continuous variables are expressed as mean ± standard deviation.

** Patients with stress urinary incontinence.

†† Measurements were performed at three anatomical ROIs, visualized on elastographic images as color-coded regions: blue = IUO, purple = MU, green = EUO (Figure 1).

p-values were calculated using the unpaired t-test or Wilcoxon rank-sum test, as appropriate.

Abbreviations: EUO = external urethral orifice; IUO = internal urethral orifice; MU = midurethra.

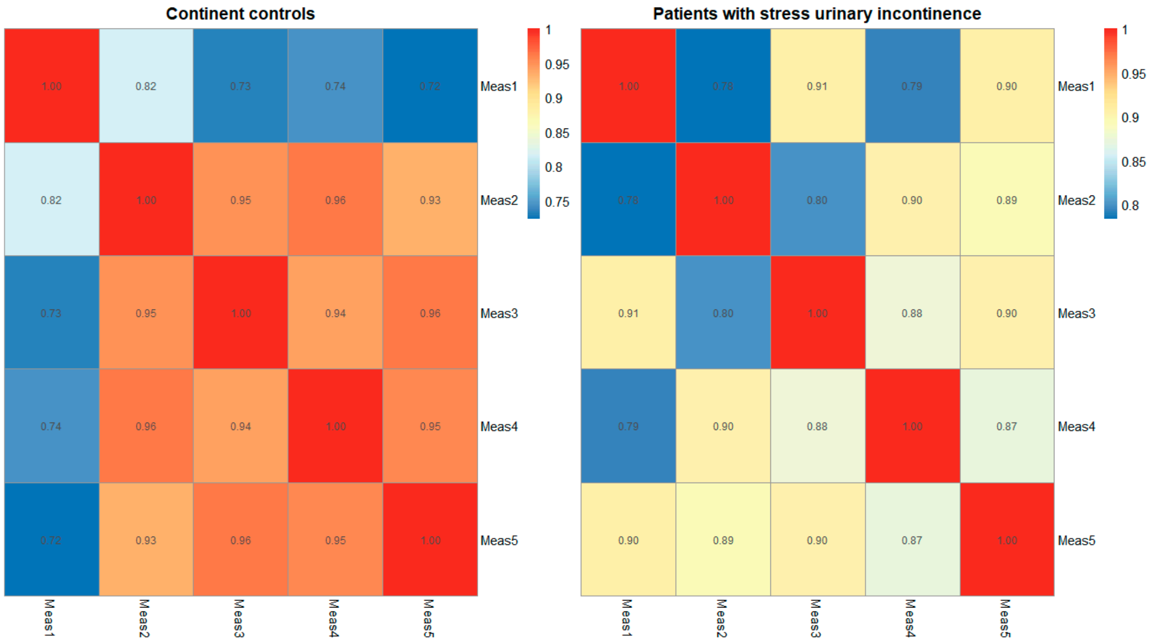

Figure S1. Intraclass correlation coefficients (ICCs) for repeated strain elastography measurements at the internal urethral orifice level, shown separately for continent controls and women with stress urinary incontinence.

Abbreviation: meas = measurement.

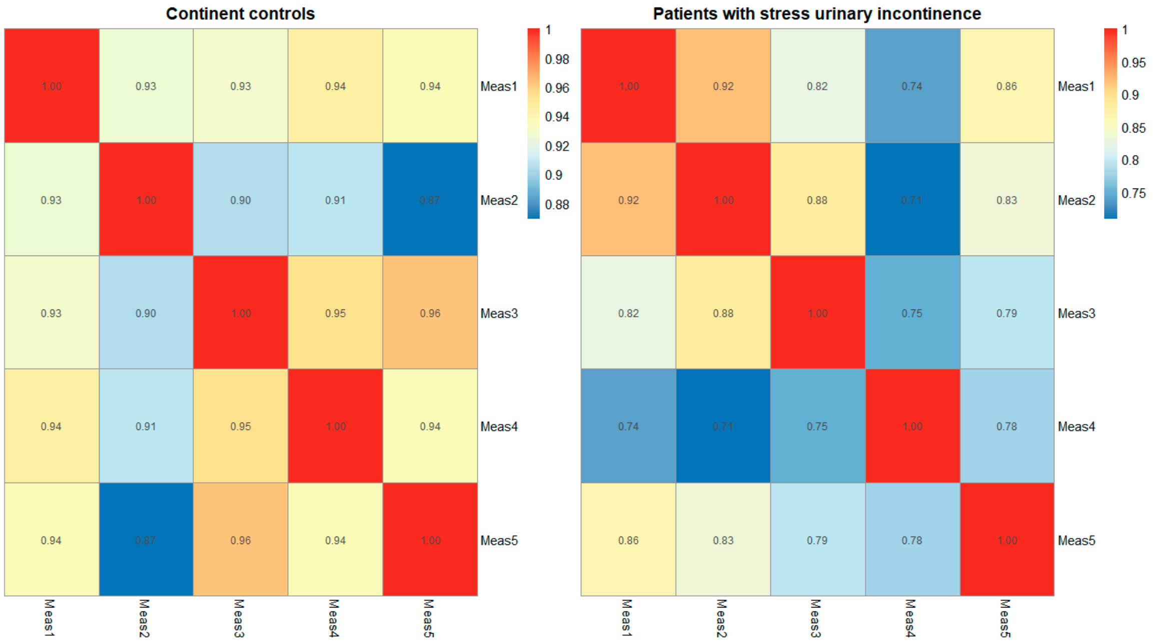

Figure S2. Intraclass correlation coefficients (ICCs) for repeated strain elastography measurements at the midurethral level, shown separately for continent controls and women with stress urinary incontinence.

Abbreviation: meas = measurement.

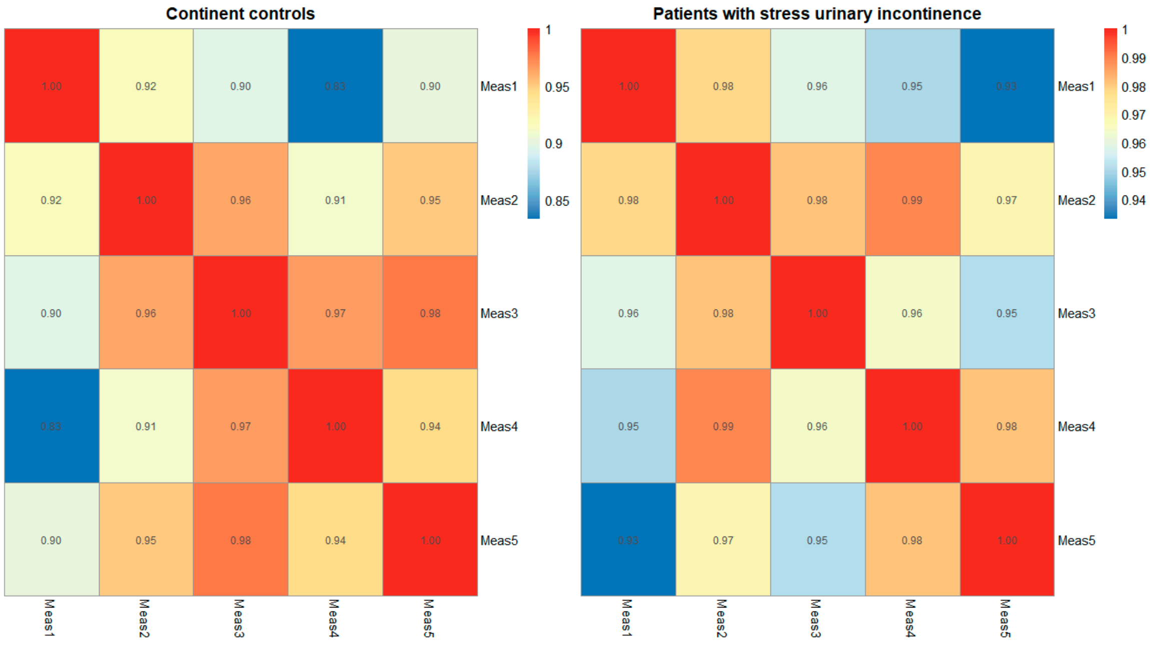

Figure S3. Intraclass correlation coefficients (ICCs) for repeated strain elastography measurements at the external urethral orifice level, shown separately for continent controls and women with stress urinary incontinence.

Abbreviation: meas = measurement.
